# Modular epistasis and the compensatory evolution of gene deletion mutants

**DOI:** 10.1371/journal.pgen.1007958

**Published:** 2019-02-15

**Authors:** José I. Rojas Echenique, Sergey Kryazhimskiy, Alex N. Nguyen Ba, Michael M. Desai

**Affiliations:** 1 Department of Organismic and Evolutionary Biology, Harvard University, Cambridge, Massachusetts, United States of America; 2 Section of Ecology, Behavior and Evolution, Division of Biological Sciences, University of California at San Diego, San Diego, California, United States of America; 3 Department of Physics, Harvard University, Cambridge, Massachusetts, United States of America; 4 NSF-Simons Center for Mathematical and Statistical Analysis of Biology, Harvard University, Cambridge, Massachusetts, United States of America; 5 Quantitative Biology Initiative, Harvard University, Cambridge, Massachusetts, United States of America; University College Dublin, IRELAND

## Abstract

Screens for epistatic interactions have long been used to characterize functional relationships corresponding to protein complexes, metabolic pathways, and other functional modules. Although epistasis between adaptive mutations is also common in laboratory evolution experiments, the functional basis for these interactions is less well characterized. Here, we quantify the extent to which gene function (as determined by a genome-wide screen for epistasis among deletion mutants) influences the rate and genetic basis of compensatory adaptation in a set of 37 gene deletion mutants nested within 16 functional modules. We find that functional module has predictive power: mutants with deletions in the same module tend to adapt more similarly, on average, than those with deletions in different modules. At the same time, initial fitness also plays a role: independent of the specific functional modules involved, adaptive mutations tend to be systematically more beneficial in less-fit genetic backgrounds, consistent with a general pattern of diminishing returns epistasis. We measured epistatic interactions between initial gene deletion mutations and the mutations that accumulate during compensatory adaptation and found a general trend towards positive epistasis (i.e. mutations tend to be more beneficial in the background in which they arose). In two functional modules, epistatic interactions between the initial gene deletions and the mutations in their descendant lines caused evolutionary entrenchment, indicating an intimate functional relationship. Our results suggest that genotypes with similar epistatic interactions with gene deletion mutations will also have similar epistatic interactions with adaptive mutations, meaning that genome scale maps of epistasis between gene deletion mutations can be predictive of evolutionary dynamics.

## Author summary

The effects of mutations often depend on the presence or absence of other mutations. This phenomenon, known as epistasis, has been used extensively to infer functional associations between genes. For example, genes that participate in the same functional module will often show a characteristic pattern of positive epistasis where the knockout of one gene will mask the deleterious effects of knockouts in the other genes. In the context of adaptation, epistasis can cause the outcomes of evolution to depend strongly on the initial genotype. Although studies have found that epistasis is common in laboratory populations, we do not know the extent to which the patterns of epistasis that reveal functional associations overlap with the patterns of epistasis that are important in evolution. Here, by comparing evolution in strains with gene deletions in different functional modules, we quantify the effect of functional epistasis on evolutionary outcomes. We find that mutants with deletions in the same module have more similar evolutionary outcomes, on average, than mutants with deletions in different modules. This suggests that screens for epistasis between gene deletion mutations will not only reveal functional interactions between those genes but may also predict evolutionary dynamics.

## Introduction

Epistasis has often been thought of as a signature of functional interactions. For example, if two residues in a protein are in physical contact, we might expect that mutations in these sites will have different effects in combination than they do independently. Similarly, if two different proteins interact, either physically or as components in some biochemical pathway, we might expect the physiological effect of knocking out both proteins together to be different from the effects of knocking out either individually.

Based on this intuition, screens for epistatic interactions have long been used as a way to identify previously unknown functional interactions. More recently, genome-wide screens of epistatic interactions among large numbers of mutations (e.g. all combinations of single-gene deletion mutations in budding yeast) have been used to characterize the global functional landscape of the cell, identifying functional modules and their interactions based on common patterns of epistasis [1–4].

Many recent studies of adaptation in laboratory microbial evolution experiments have shown that epistasis among adaptive mutations is relatively common and widespread. For example, several studies reconstructed all possible combinations of mutations that accumulate along the line of descent in adapting populations, finding that epistasis is pervasive [5–7]. Other studies have used patterns of evolution across multiple replicate lines to infer patterns of epistasis [8–14]. For example, Tenaillon et al. [15] found that epistasis within specific functional modules leaves clear signatures in the patterns of parallel evolution across 114 replicate *Escherichia coli* populations adapting to high temperature.

However, many of these laboratory evolution studies have not identified a clear connection between the patterns of epistasis among adaptive mutations and functional interactions. For example, numerous studies have found that epistasis among beneficial mutations is generally negative (i.e. double-mutants are less fit than the product of the single-mutant fitnesses) [6, 7, 10, 14, 16], though there are a few exceptions [17]. This trend towards negative epistasis is one explanation for the fact that these experiments tend to show a general pattern of declining adaptability: initially more-fit populations tend to adapt more slowly than less-fit populations [18].

By reconstructing sets of adaptive mutations in a variety of genetic backgrounds, several studies have argued that this generally negative epistasis reflects an overall pattern of diminishing returns epistasis, where the fitness effect of any individual beneficial mutation tends to decline systematically with the fitness of the genetic background on which it occurs [6, 7, 10]. This picture of a global pattern of diminishing returns epistasis stands in contrast to the typical view of epistasis as a marker of functional interactions. However, diminishing returns has primarily been observed among small sets of mutations that arise in laboratory evolution experiments, which each start with a specific initial strain that adapts to a specific environmental condition. To the extent that this initial strain is poorly adapted to that environment due to a particular type of defect, all subsequent adaptation may reflect ways to correct this defect. This could lead to sets of adaptive mutations that reflect one or a few functional pathways and hence interact in a way that does not reflect the overall functional landscape of the cell. In other words, if the environment and initial genotypes select for adaptive mutations that are all within one or a few specific modules, the patterns of epistasis among these mutations will reflect only these modules and not the overall organization of the cell.

To find more general signatures of functional epistasis, it might thus be useful to compare the evolutionary fates of initial strains that have a variety of different types of defects. With this in mind, a few recent studies have investigated adaptation among diverse initial genotypes. For example, Jerison et al. [12] studied how initial genotype affects the rate and genetic basis of adaptation among 230 offspring of a cross of two distantly related yeast strains. Similarly, Szamecz et al. [11] analyzed compensatory adaptation in 187 yeast strains, each with a different single gene knockout. Remarkably, these studies both found that the rule of declining adaptability still applies across these diverse initial genotypes. It is possible that, despite being more diverse, these initial genotypes still share a common defect that drives adaptation. However, since neither study reconstructed individual mutations in different genetic backgrounds, it is unclear whether the rule of declining adaptability in these experiments arises due to general diminishing returns epistasis. Instead, there are hints of other factors involved in both cases. For example, Szamecz et al. [11] found that the mutations acquired in each strain are enriched in genes that share functional annotations with the deletion in that initial strain.

Here, we sought to investigate the relationship between the functional landscape of yeast (based on genome-wide screens for epistasis among deletion mutants) and the patterns of epistasis among mutations that arise during laboratory adaptation. To do so, we analyze compensatory adaptation in 37 strains, each founded by a single gene deletion mutant nested in one of 16 functional modules (plus a negative and positive control) defined using the yeast genetic interaction map [3]. We evolved 20 replicate lines founded by each of these 37 deletion mutants, finding that the overall rule of declining adaptability still applies—initially less-fit strains tend to adapt more rapidly than more-fit strains. However, we also find signatures of the functional landscape: strains with deletions in the same module tend to adapt more similarly than those with deletions in different modules, even after accounting for the effects of declining adaptability.

To investigate the patterns of epistasis underlying these results more directly, in a subset of lines we reconstructed evolved mutations on a wild-type background lacking the initial gene deletion. We find that many mutations do not reflect compensation for the specific functional defects introduced by the initial deletion. Instead, they are adaptive in both wild-type and deletion backgrounds. However, this is not universal: some evolved mutations do compensate for defects that are specific to the initial deletion in that line. This compensatory adaptation results in patterns of epistasis that can lead to evolutionary entrenchment of the original deletion.

## Results

We began by choosing 43 gene deletion mutants, selected to reflect a few examples from each of a range of distinct functional categories (see Methods for details on how these were chosen). Using data from the yeast genetic interaction map (generated from a genome-wide screen of epistasis among gene deletion mutants [3]), we assigned these 43 gene deletion mutants to 16 different functional modules (Table 1), each with highly correlated intra-module genetic interaction profiles (with mean between gene Pearson correlations ranging from 0.21 to 0.72, S1 Table). We also chose one deletion each to represent a negative and positive control. From a common ancestor, we attempted to create one strain carrying each of these gene deletion mutations (see Methods for details on strain construction). We were able to successfully construct and verify 37 of the 45 deletion mutants we attempted; note that this left some modules with only a single gene to represent them. We measured the fitness of each of these 37 strains, finding that the initial deletions have a wide range of fitness effects (from −24% to 4% per generation; see Table 1).

**Table 1:** Fitness gain by gene deletion mutant.

Module	Gene	Initial fitness (%)	Fitness gain (%)
Central metabolism 1	*hxk2*Δ	−24.5	13.4 ± 2.9
	*tps1*Δ	−24.4	21.8 ± 2.3
Central metabolism 2	*lat1*Δ	−13.9	9.2 ± 2.4
	*pda1*Δ	0.1	3.3 ± 1.2
	*pdb1*Δ	−15.5	9.7 ± 2.7
Electron transport chain	*coq2*Δ	−4.6	4.3 ± 1.5
	*cox14*Δ	−5.2	4.5 ± 1.3
	*cox6*Δ	−9.8	6.5 ± 1.0
Elongator complex	*elp4*Δ	−14.0	2.7 ± 1.1
	*elp6*Δ	−14.2	2.6 ± 0.9
	*kti12*Δ	−12.2	3.6 ± 1.3
	*ncs2*Δ	−13.1	2.6 ± 0.4
	*uba4*Δ	−13.8	3.3 ± 0.6
Golgi/endosome/vacuole 1	*get1*Δ	−7.3	3.4 ± 1.4
	*get2*Δ	−10.4	9.1 ± 1.0
Golgi/endosome/vacuole 2	*pep8*Δ	−1.6	3.6 ± 1.3
	*vps29*Δ	−1.9	3.3 ± 1.1
	*vps35*Δ	−1.9	2.4 ± 1.0
Kinetochore	*chl4*Δ	−0.5	2.2 ± 0.6
	*ctf19*Δ	−1.1	1.1 ± 0.8
	*iml3*Δ	−0.4	2.5 ± 0.8
Morphogenesis	*arc18*Δ	3.9	−0.0 ± 1.4
	*she4*Δ	−20.4	7.0 ± 3.1
Nuclear migration	*arp1*Δ	−3.0	5.4 ± 0.5
	*nip100*Δ	−8.7	8.2 ± 1.4
Peripheral metabolism	*erg3*Δ	−6.3	1.4 ± 1.4
	*erg5*Δ	−1.7	3.2 ± 2.2
	*erg6*Δ	−6.8	1.9 ± 1.7
RNA processing	*lsm1*Δ	−10.1	2.0 ± 0.8
	*pat1*Δ	−13.5	4.6 ± 0.5
Ras signaling 1	*bmh1*Δ	−3.4	6.4 ± 1.5
Ras signaling 2	*ira2*Δ	−4.4	7.8 ± 1.0
Nuclear pore	*nup2*Δ	−2.3	4.1 ± 0.7
Stress response 1	*sfl1*Δ	−1.3	4.1 ± 0.9
Stress response 2	*sok2*Δ	−1.9	4.8 ± 0.8
Negative control	*ho*Δ	−0.0	3.4 ± 1.3
Positive control	*ade2*Δ	−23.0	16.7 ± 1.5

Initial fitness of all 37 gene deletion mutant founders and the mean and standard deviation in fitness gain (relative to that founder) of the 20 replicate populations descended from each.

### The rate of adaptation

**Fig 1:**
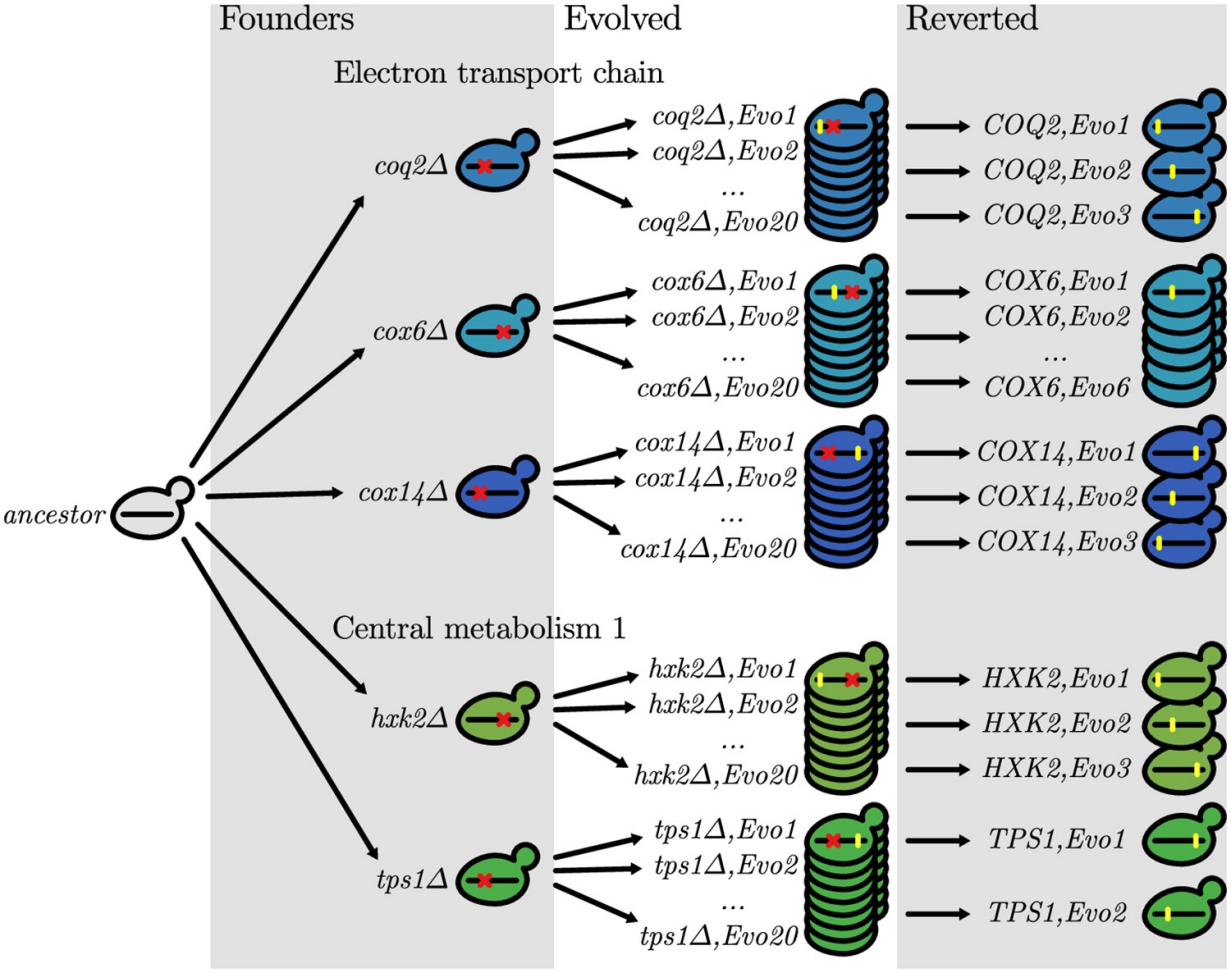
Experimental design showing genotypes of important strains. Schematic showing 5 of the 37 gene deletion founders, from 2 of the 16 different functional modules studied here (see Table 1 for the full list). After constructing each gene deletion founder, we founded 20 replicate populations from each and allowed them to evolve for 500 generations. Finally, we reverted the initial gene deletion in a subset of clones from these evolved populations.

**Fig 2:**
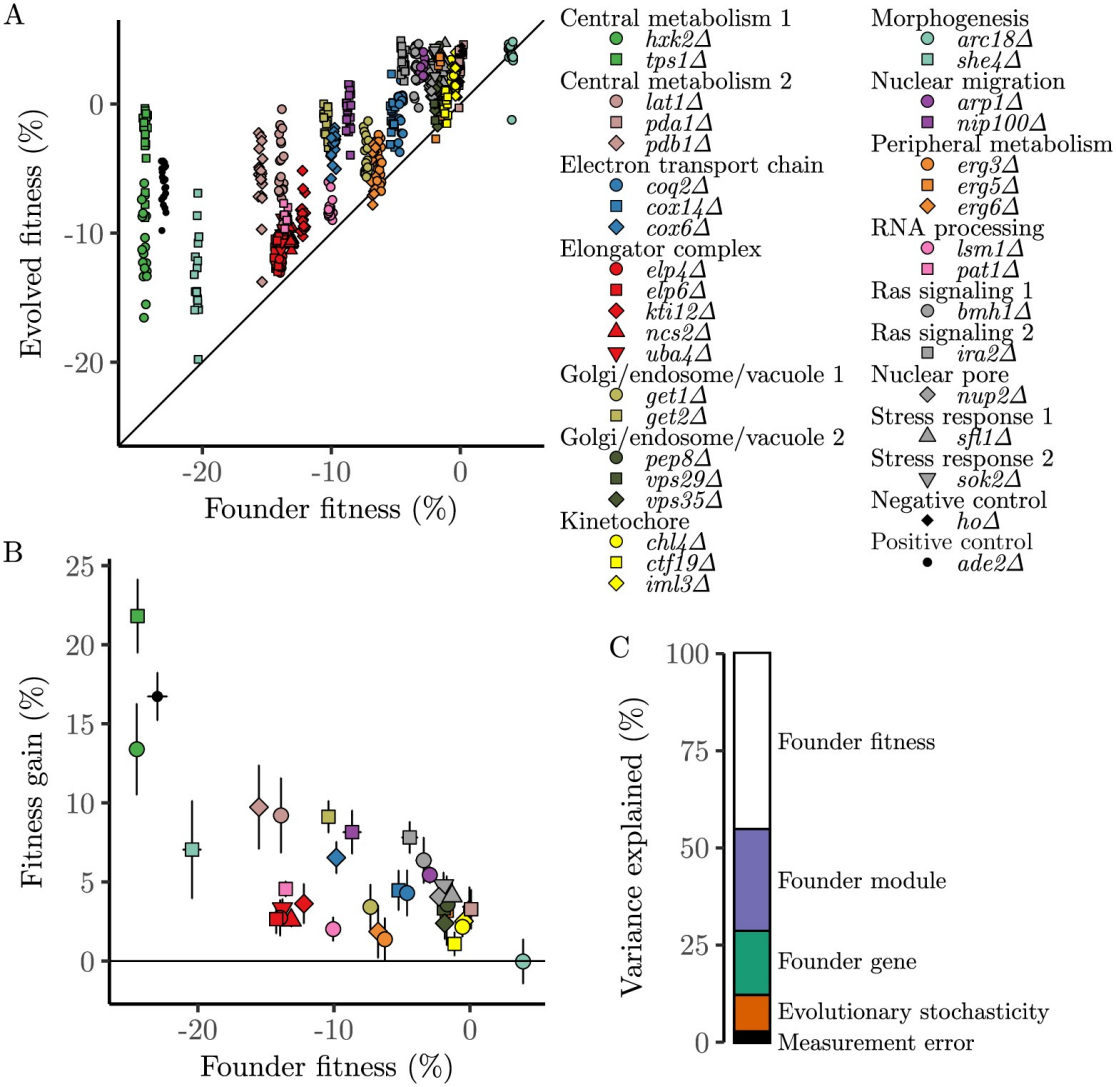
Fitness evolution. (A) Relationship between the initial fitness of the 37 Founder gene deletion mutants and the fitness of each of the 20 replicate populations after 500 generations of evolution, colored according to functional module. The *x*-coordinate is jittered slightly for visibility. (B) Relationship between initial fitness of the 37 Founder gene deletion mutants and the mean fitness gain of the 20 replicate populations descended from that Founder after 500 generations of evolution. Horizontal error bars show standard errors of the mean initial fitness and vertical error bars show standard deviations in the fitness of descendant populations. (C) Fraction of the variance between populations in fitness gain after 500 generations of evolution that is attributable to each indicated component. All variance components are significant (Table 3).

We founded 20 replicate populations from each of the 37 gene deletion mutants, and evolved the resulting 740 populations in batch culture in rich laboratory media (YPD) at an effective population size of about 5 × 10^4^ using our standard methods for laboratory evolution experiments (Fig 1, Methods). After 500 generations, we measured the fitness of each evolved population. In Fig 2A we show how the fitness of each evolved line depends on the initial fitness of the gene deletion mutant from which it descends (the “Founder fitness”). We see that most evolved lines increased in fitness, though there is substantial variation in the extent of this adaptation even among lines descended from the same Founder. In Fig 2B we show how the average fitness gain in lines founded from each Founder depends on that Founder fitness. We see that there is a general trend of declining adaptability: lines descended from less-fit Founders tend to adapt more rapidly than those descended from more-fit Founders. However, there is also substantial variation between Founders, with some Founders adapting systematically more or less rapidly than others at similar initial fitness.

There are several sources of variation in the rate of adaptation across our 740 evolved populations. We are primarily interested in the effect of the Founder genotype: that is, how the initial gene deletion influences the rate and genetic basis of adaptation in its descendant lines. In addition to the effect of Founder genotype, the evolutionary process is inherently random, so we expect some inherent evolutionary stochasticity in how rapidly any evolved population evolves. This inherent stochasticity leads to variation between lines descended from the same Founder genotype. Further, measurement error in our fitness assays leads to additional variation, which we can quantify by comparing replicate measurements of the same evolved populations.

To quantify the relative importance of these different factors, we conducted a hierarchical analysis of variance to partition the variation in the rate of adaptation between our evolved lines into the components that can be attributed to Founder identity, evolutionary stochasticity, and measurement error (Fig 2C, Methods). We find that Founder identity plays a dominant role, explaining 88 percent of the variance in rate of adaptation, while inherent evolutionary stochasticity explains 9 percent and measurement error explains 3 percent. These latter two effects lead to similar absolute amounts of variance as reported by Kryazhimskiy et al. [10] in a study analyzing the rate of adaptation in lines descended from a set of very closely related Founder genotypes. However, in the present study this corresponds to a much lower fraction of variance, because we find much greater overall variation in evolutionary outcomes and this additional variance is almost entirely explained by Founder genotype.

We can further subdivide the effect of Founder genotype into several components. We find that the initial Founder fitness can explain almost half (46 percent) of the total variance in the rate of adaptation. As we will describe below, this fitness effect is consistent with a rule of declining adaptability, where initially less-fit strains adapt more rapidly than initially more-fit strains. Above and beyond this effect of fitness, the module identity in which the initial gene deletion is categorized (“Founder module”) explains 26 percent of the variance. Finally, the idiosyncratic effect of the specific gene deletion, above and beyond the effect of module and fitness, contributes an additional 16 percent of the variance (Fig 2C).

### Epistatic signatures of compensatory adaptation

We next sought to more directly investigate the underlying patterns of epistasis that lead to the effects of Founder genotype on the rate of adaptation. To do so, we attempted to revert the initial Founder gene deletion in each of 270 evolved lines (about 7 lines descended from each of the 37 Founder genotypes). Specifically, we used standard transformation methods on whole-population samples from each of the 270 evolved lines, and selected three independent transformants from each (Methods).

This procedure led to three revertant clones from each of 100 independently evolved populations. In the remaining 170 populations, we were unable to obtain revertant transformants. The populations in which we were able to obtain reversions were distributed in a highly nonrandom way across Founder genes and Founder modules (Fisher’s exact test, *p* ≪ 0.01 in both cases, S2 Table); in six cases all evolved descendants of a given Founder genotype produced no transformants.

In addition to experimental errors, there are two potential explanations for this phenomenon. The first possibility is that these evolved populations share mutations that make them less transformable or change the copy number of the deletion cassette. Either of these changes would disrupt our method for making the reversions (see Methods). One potential candidate for such a mutation would be a spontaneous autodiploidization event, which has been observed to occur frequently in other yeast evolution experiments [19–21]. The other possibility is strong epistasis: compensatory mutations in evolved clones are lethal or very strongly deleterious in the absence of the initial deletion. To be conservative in inferring effects of functional module, we excluded from further analysis nine Founder genotypes from which we were not able to generate revertant transformants in at least two independently evolved descendant populations (specifically, we excluded 2 populations descended from founders that only yielded a single revertant, leaving a total of 98 populations for further analysis). The remaining 28 founders have a uniform rate of reversibility (about 54 percent).

We measured the fitness of each of the 294 revertant clones (3 clones derived from each of 98 evolved populations descended from 28 different Founder genotypes). In almost all cases, the three revertant clones descended from the same evolved population had very similar fitness, with a few outliers likely caused by mutations either segregating in the evolved populations or introduced due to transformation artifacts (S1 Fig). We use the median fitness of the three clones derived from a given evolved population as a measure of the fitness effect of the mutations that accumulate during adaptation of that line (the “evolved mutations”) on the wild-type ancestral background. In contrast, the fitness of the evolved line minus the fitness of the initial Founder gene deletion it descends from reflects the fitness effect of the evolved mutations on the background of the initial Founder gene deletion. The difference between these two fitness effects, denoted by *ϵ*, indicates epistasis between the evolved mutations and the initial gene deletion.

**Fig 3:**
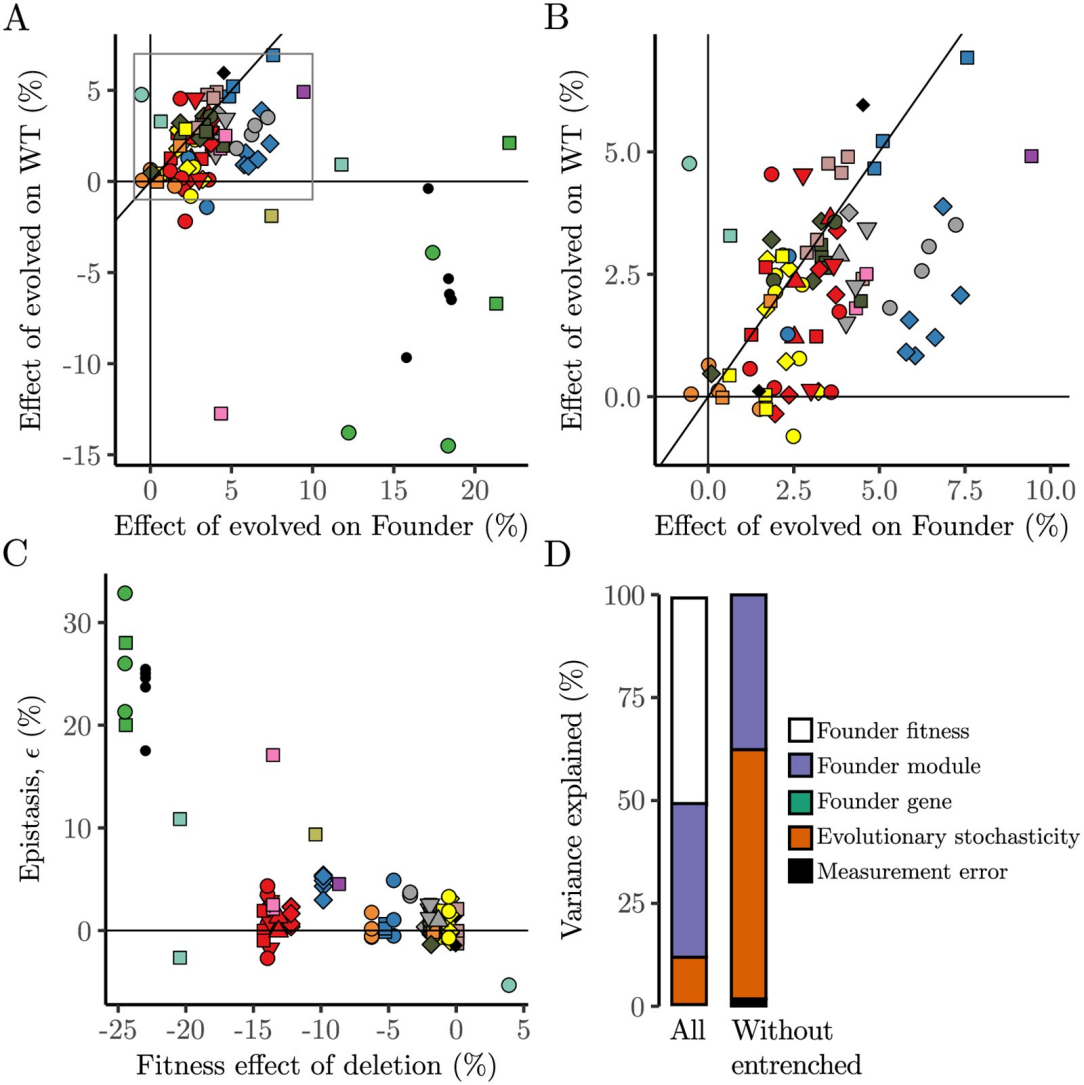
Epistasis between Founder gene deletion and evolved mutations. (A) Relationship between the fitness effects of evolved mutations in the Founder deletion background compared to their effect in the wild type background. Boxed region shows area expanded in (B). (C) Epistasis, *ϵ*, between evolved mutations and Founder deletion mutation plotted against the fitness effect of Founder deletion mutation. Refer to Fig 2 for the symbol legend. (D) Fraction of the variance between populations in epistasis between Founder gene deletion and evolved mutations that is attributable to each indicated component. The analysis was performed with all reverted populations (All) and repeated excluding descendants of *ade2*Δ, *hxk2*Δ and *tps1*Δ (Without entrenched). All variance components are significant (Table 4).

In Fig 3A we show how the fitness effects of the evolved mutations on the ancestral background depend on their fitness effects on the background of the initial gene deletion in which they evolved. In the majority of cases (see Fig 3B), we see that the evolved mutations are moderately beneficial in both backgrounds, with an enrichment for cases where the evolved mutations are more strongly beneficial in the deletion background than the wild-type background. In principle, since the initial deletion backgrounds are typically less fit than the wild-type ancestor, this could reflect diminishing returns epistasis. However, in Fig 3C, we show that (with a few exceptions we return to below), the fitness of the initial gene deletion mutant does not strongly affect *ϵ*, the difference between the fitness of the evolved mutations in the deletion versus wild-type background. Thus, the enrichment of positive epistasis between evolved mutations and initial gene deletions may reflect functional coupling where some of these evolved mutations provide compensatory adaptation to the defects introduced by the initial gene deletions.

We further sought to quantify the extent to which these epistatic differences (as measured by *ϵ*) depend on the Founder identity. To do so, we conducted a hierarchical analysis of variance to partition the variance in *ϵ* into contributions from measurement error, evolutionary stochasticity, and Founder identity (including fitness, module, and gene). We find that while evolutionary stochasticity plays a large role, there is some effect of Founder identity (Fig 3D). Thus, some initial founding gene deletions are more likely to lead to specific compensatory adaptation.

### Entrenchment

As described above, in the majority of cases, evolved mutations were beneficial in both the ancestral and evolved backgrounds. However, there were a few exceptions. Most strikingly, in all evolved lines descended from Founder genotypes with deletions in one module (*hxk2*Δ and *tps1*Δ in the Central metabolism 1 module (Table 1)) and in *ade2*Δ (our positive control for functionally dependant adaptation (see below)), the evolved mutations were strongly beneficial in the background of the initial gene deletion in which they evolved but strongly deleterious in the ancestral background.

**Fig 4:**
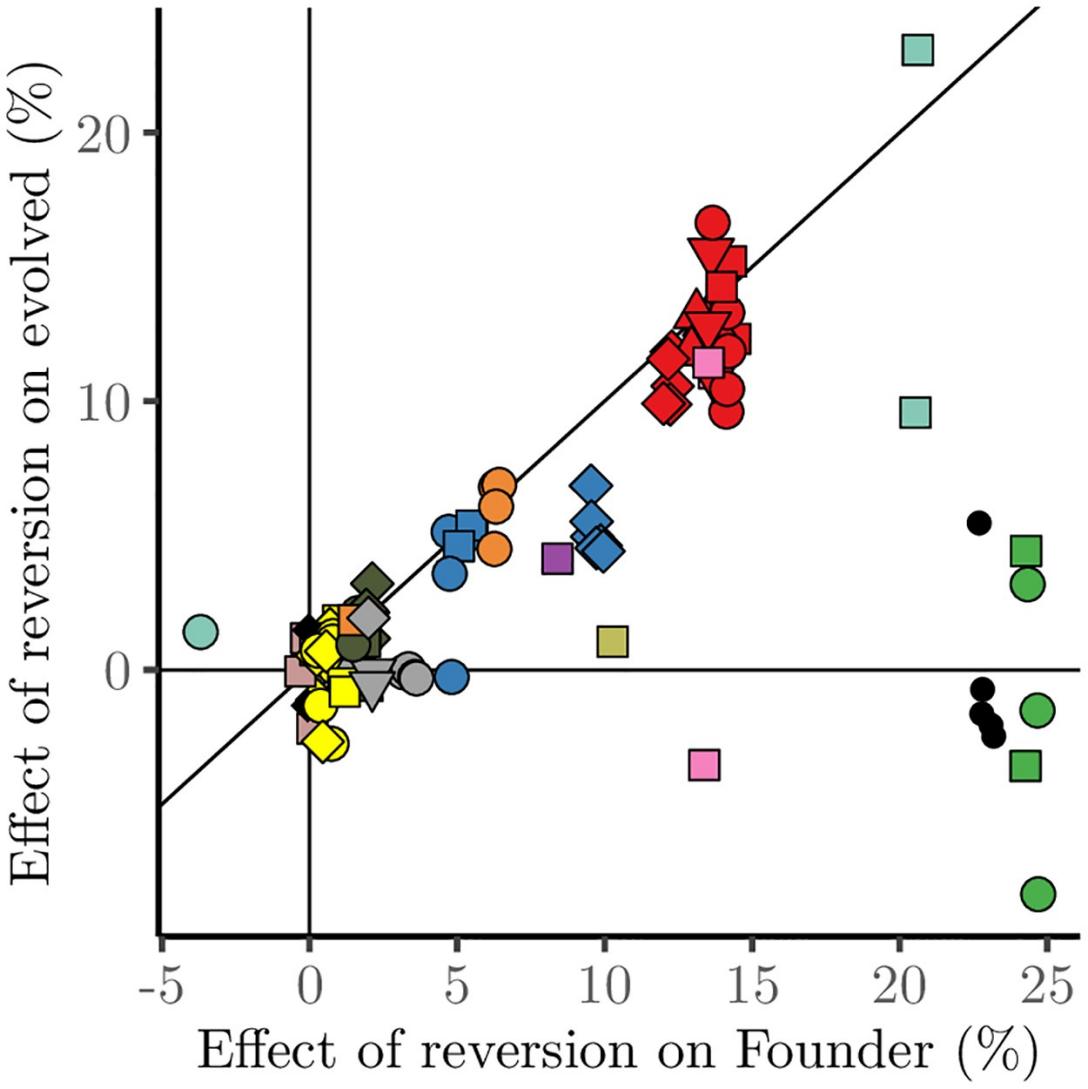
Effects of reverting founding gene deletion mutations. Fitness effects of reverting the initial gene deletion mutations in the evolved background plotted against the fitness effect of reverting the initial gene deletion in the wild-type background. Refer to Fig 2 for the symbol legend.

These cases can lead to evolutionary entrenchment of a deleterious gene deletion: although it would have initially been favorable to revert the deletion, after compensatory evolution this is no longer beneficial (Fig 4). In other cases, despite the fact that the evolved mutations are deleterious in the ancestral wild-type background, reverting the initial deletion is still beneficial (though less so than before compensatory adaptation).

For lines descended from the *ade2*Δ Founder genotype, the mechanism for evolutionary entrenchment is straightforward. Briefly, *ADE2* codes for an enzyme in the adenine biosynthesis pathway immediately downstream of a toxic metabolic intermediate (S2 Fig). Thus the *ade2*Δ mutation is strongly deleterious because it leads to accumulation of the toxic intermediary. In this genetic background, loss-of-function mutations in genes that code for enzymes upstream of ADE2 in the adenine biosynthesis pathway will eliminate the precursors to this toxic intermediary and hence compensate for the deleterious effect of the initial *ade2*Δ mutation. However, these upstream loss-of-function mutations are deleterious in the ancestral background because they eliminate adenine biosynthesis. We sequenced five populations descended from the *ade2*Δ Founder (Methods), and found that, consistent with this expectation, all five populations had a loss-of-function mutation in a gene upstream of *ADE2* (either *ADE4*, *ADE6*, or *ADE8*; one population acquired mutations in both *ADE4* and *ADE8*).

In contrast to the case of *ade2*Δ, the mechanism of compensation and entrenchment in lines descended from the *hxk2*Δ and *tps1*Δ Founders is unclear. We sequenced three populations descended from the *hxk2*Δ Founder, finding that two had mutations in *ATP2* and one had a mutation in *ATP1*. These genes code for components of the mitochondrial ATP synthase complex. While both *HXK2* and the ATP synthase are crucial components of central carbon metabolism in yeast, we lack a mechanistic explanation of how perturbations to the ATP synthase could compensate for the deletion of *HXK2*. One possibility is that because our *hxk2*Δ founder has impaired mitochondrial function (as indicated by its reduced ability to grow on the glycerol media), mutations in *ATP1* and *ATP2* may compensate for a loss of membrane potential in the mitochondria. van Leeuwen et al. [22] found mutations in *ATP1* and *ATP2* in strains lacking mitochondrial DNA (including one specific mutation, ATP2-Q412E, also found in our evolved lines) and suggested that these mutations may reverse ATP synthase activity to generate ADP3^−^ instead of ATP4^−^ and thereby allow the mitochondria to rebuild a membrane potential, which is thought to be required for protein import into the mitochondria. We also sequenced two populations descended from the *tps1*Δ Founder but were unable to identify any of the compensatory mutations (Methods). This may simply reflect the limited coverage of our sequencing, though given the above discussion it is also possible that compensation in this case involves mitochondrial mutations.

### Determinants of the genetic basis of adaptation

We next sequenced one clone from each of the 100 evolved populations in which we were able to successfully revert the initial gene deletion. We used the breseq software package [23] to identify a total of 153 coding mutations across the 100 sequenced clones (Methods); a list of all coding mutations called in each clone is given in Supplementary S2 Data. We note that because our sequencing method is unable to identify certain types of mutations (e.g. mitochondrial mutations and large indels and structural variants, see Methods), this represents only a subset of all mutations present in these clones. Keeping this caveat in mind, we do see a weak relationship between the number of mutations in each evolved line and the initial fitness of the founding genotype as well as with the fitness gain during compensatory adaptation (S7 Fig). Thus larger fitness increases during compensatory adaptation may result from more compensatory mutations (though it may also be true that the compensatory mutations that do occur have larger fitness benefits).

**Fig 5:**
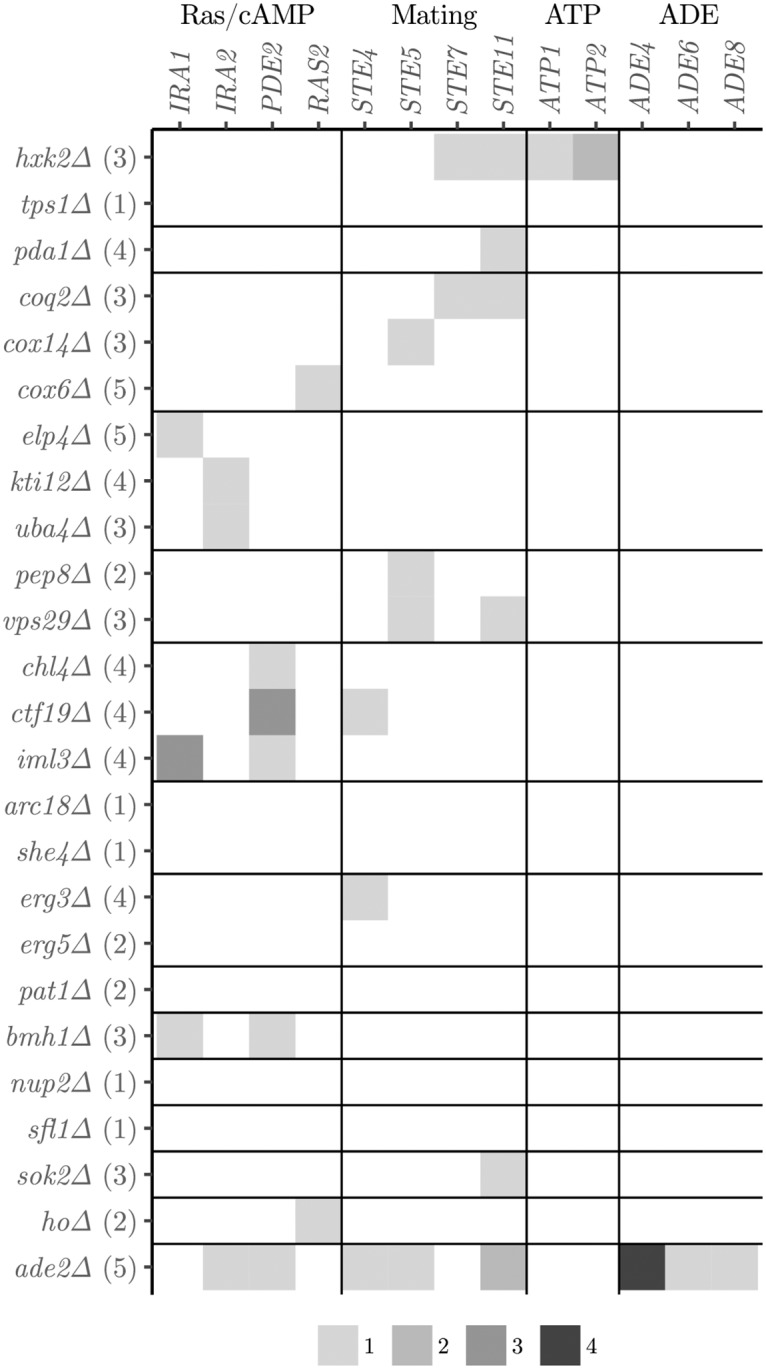
List of multi-hit mutations. Genes that were independently mutated in at least two different populations by Founder gene deletion (number of populations analyzed), grouped by mutation module and Founder module.

To analyze how Founder identity and other factors influence the genetic basis of adaptation, we analyzed the patterns of parallelism in acquired mutations. We first investigated parallelism at the gene level, focusing on genes that were independently mutated in at least two populations (Fig 5) because genes mutated only a single time provide no additional power in the gene-level analysis. These “multi-hit” genes are likely to be enriched for beneficial mutations that were drivers of adaptation in that population. Excluding the descendants of the *ade2*Δ and *hxk2*Δ founders, we found that all multi-hit genes belong to pathways that are common targets of laboratory adaptation, the Ras/cAMP and the mating pathways [10, 12, 20, 24]. This is unsurprising, because highly specific compensatory mutations for particular initial Founder gene deletions would be beneficial in only a small number of evolved lines, and hence much less likely to appear as multi-hit genes. Instead, it is likely that these multi-hit genes represent mutations that are generally adaptive to the laboratory conditions in our system.

Nevertheless, it is possible that initial gene deletions in different modules could enrich for particular sets of adaptive mutations. This is clearly true in the descendants of the *ade2*Δ and *hxk2*Δ Founders. To measure the potential influence of other Founder genotypes on the identity of the multi-hit genes accumulated in its descendants, we calculated the mutual information between mutated genes and Founder modules (excluding *ade2*Δ and *hxk2*Δ) and compared it to a null distribution obtained by permuting the data while keeping the number of mutations per clone fixed (Methods). Intuitively, the mutual information measures the amount of information (in bits) that we gain about the identities of mutated genes by knowing the identity of the Founder. Higher values of mutual information indicate that the Founder identity more strongly predicts the genes that are mutated.

We found a significant relationship between acquired mutations and functional module of the initial Founder gene deletion (Table 2, top left). To measure the additional information provided by the specific Founder gene deletion (after controlling for the effect of the functional module corresponding to this gene), we performed an analogous analysis to compute the mutual information between Founder gene and acquired mutations, conditioning on Founder module. We found a significant association between acquired mutations and the Founder gene even after conditioning on Founder module (Table 2, bottom left). These results indicate that even among apparently generally adaptive multi-hit genes, the Founder genotype does influence the genetic basis of adaptation in a module- and gene-specific way (i.e. descendants of Founders with deletions in the same module are more likely to acquire mutations in the same multi-hit genes).

**Table 2:** Mutual information between founder genotypes and evolved mutations.

	Evolved genes		Evolved interaction clusters	
	$M-{\bar{M}}_{p}$ (95% CI)	*p*-value	$M-{\bar{M}}_{p}$ (95% CI)	*p*-value
Founder module	0.25 (0.05, 0.36)	0.011	0.29 (0.13, 0.43)	< 0.001
Founder gene	1.21 (0.45, 1.81)	< 0.001	0.91 (0.17, 1.54)	0.010

Mutual information (in bits) in excess of null for founder modules with evolved genes or evolved interaction clusters and for founder genes with evolved genes or evolved interaction clusters, conditioning on founder module.

We next analyzed patterns of parallelism at the level of functional modules, focusing now on clusters defined by genetic interactions, “interaction clusters”, (see Methods) that were independently mutated in at least two populations (S3 Table). We repeated our mutual information analysis at this level, finding statistically significant associations between the Founder genotype (both module and the specific gene deletion) and the interaction cluster in which mutations arose in its descendants (Table 2, right column).

## Discussion

Large-scale surveys of epistatic interactions have long been used to investigate the functional organization of cellular processes. This approach has been used to reveal the binding structure of members in protein complexes, the biochemical order of enzymes in metabolic pathways, the interactions between different complexes and pathways, and the relationships between biological process at the highest levels of cellular organization [3, 25, 26]. We expect these patterns of epistasis to also have important consequences for the rate and molecular basis of adaptation, and to influence the degree of parallelism and contingency in evolving populations [27–30]. For example, we might expect functional epistasis to lead to historical contingency that decreases the degree of parallelism in evolution, as different lines stochastically accumulate mutations in different functional modules and then tend to accumulate different compensatory mutations in future adaptation. However, the connection between the epistatic signatures of functional modules and the patterns of epistasis important to evolutionary dynamics have not been extensively studied. It is thus unclear how observations of the effects of epistasis in evolutionary dynamics can be predicted from (or be used to infer) functional organization.

In many earlier laboratory evolution experiments, the most striking pattern of epistasis between adaptive mutations is a general tendency towards negative interactions (e.g. global diminishing returns epistasis [10]). These studies suggest that, with a few exceptions, the bulk of the interactions between mutations that are relevant for adaptation in these systems can be explained without any functional information using a simple model of diminishing returns, in which the fitness effect of a beneficial mutation is systematically smaller in higher-fitness genetic backgrounds. However, other laboratory evolution studies have found some signatures of epistatic interactions that reflect functional organization [11, 12, 15].

Here, we describe an experiment designed to test the degree to which functional relationships, as defined by a genome-wide screen of epistatic interactions, influence evolutionary dynamics. Our hierarchical design, in which we evolved 20 replicate lines descended from each of 37 gene deletion mutants representing 16 functional modules (plus two controls), allows us to quantify the effects of gene and module identity on the rate and genetic basis of adaptation. We find that the rule of declining adaptability still applies in this system, and initial fitness can explain almost half of the variation in the rate of adaptation of different strains. The mechanistic basis of this effect of initial fitness remains unclear. However, in addition to this effect, we find that functional epistasis does indeed have predictive power: populations descended from Founders with gene deletions in the same functional module adapted more similarly than average, even after controlling for the effects of initial fitness.

In a few cases, the functional basis for this pattern was straightforward: strong epistatic interactions between the initial gene deletions *ade2*Δ and *hxk2*Δ and the mutations in their descendant lines indicated a clear functional interaction. In other cases, while some form of epistatic interactions between acquired mutations and the initial deletion leads to a signature of similarity between lines descended from the same Founder (and Founders with deletions in the same module) the functional basis of these effects is less clear.

It is important to note that our experimental approach has several important limitations. One key limitation is the time scale of our experiment: our analysis of evolutionary outcomes after 500 generations can only give a snapshot of a phenomenon that is likely to be much richer. Second, our study was carried out in a single strain background, BY4741 (very similar to the strain background used in Costanzo et al. [3]; the only differences are that our strains are uracil and methionine prototrophs), and a single environment, rich laboratory yeast media. Since the function and typical effect size of generally adaptive mutations is different in different strains and environments, it would be surprising if the effect of functional module on compensatory adaptation did not depend on the strain background or environment used for evolution. However, we note that an earlier study by Szamecz et al. [11] measured the adaptation of 4 replicates of each of 187 yeast deletion strains in a closely related genetic background. While these authors did not employ the same type of hierarchical design, we can apply the same analysis framework that we have used here, and find that consistent with our results, compensatory adaptation in this earlier study is affected both by initial fitness and by functional module (S8 Fig).

Despite these limitations, our results show that the functional information revealed by genome scale maps of epistasis between gene deletion mutations is indeed predictive of evolutionary dynamics, at least in our system. Thus, genotypes with similar epistatic interactions with gene deletion mutations also seem to have similar epistatic interactions with adaptive mutations. These interactions can have important long-term evolutionary impacts, affecting patterns of parallelism and repeatability. For example, we found several cases where evolution leads to the entrenchment of initially deleterious gene deletions. This entrenchment can lead to extensive historical contingency in adaptive trajectories, potentially driving irreversible divergence between populations [27, 30, 31], though we note that weaker forms of epistasis can also lead to similar contingency, particularly when clonal interference is important [32]. While we have identified only a few cases of entrenchment here, it is important to note that we were unable to generate reversions of the initial Founder gene deletions in a number of cases. We therefore cannot rule out the possibility that these cases may reflect even more extreme forms of entrenchment, where reverting the initial deletion becomes lethal after compensatory adaptation.

Our results also highlight how laboratory evolution experiments could be useful as a way to investigate the functional organization of the cell. Large-scale hierarchically organized experiments of the type we describe can in principle be used as a type of screen for epistatic interactions that might have more subtle or undetectable effects using other methods (e.g. in direct genome-wide gene deletion screens [1–4, 33] or suppressor screens [22, 34–36]), or might involve types of mutations that are difficult to screen via other methods. The patterns of parallelism between replicate lines could then be used to create a type of evolutionary similarity metric which could be the basis for an alternative functional clustering.

## Methods

### Assigning genes to functional modules

Our ability to measure the effect of functional epistasis on the rate of adaptation depends on assigning genes to functional modules. To do so, we relied both on curated functional annotations and on the yeast genetic interaction map. Annotations can be used directly to group genes into protein complexes, metabolic or signaling pathways, and broader biological process. On the other hand, the genetic interaction map [3] does does not assign genes to distinct groups. Instead, the interaction map, which consists of measurements of epistatic interactions between about 23 million pairs of gene deletion mutations in yeast, provides a genetic interaction profile for each gene, which shows how it interacts with other genes in the genome. Costanzo et al. [3] argued that correlated interaction profiles imply close functional relationships and used this insight to infer the modular organization of the yeast genome. We reasoned that groups of genes with both functionally related annotations and correlated genetic interaction profiles would be most likely to exhibit signatures of functional epistasis.

We first clustered all genes using correlations in their genetic interaction profiles as a similarity metric. Specifically, we performed hierarchical clustering using Ward’s clustering criterion (implemented in the R function hclust with option ward.D2 [37]) on a matrix of gene-gene distances defined for each pair of genes as 1 − |*ρ*|, where *ρ* is the Pearson correlation in genetic interaction profiles of the two genes. We then filtered out genes without significant fitness effects, genes of unknown function, and genes known to increase mutation rate or cause other genetic instabilities. From the remaining set of clustered genes, we compared cluster membership to gene annotations and hand selected 43 genes from 16 different clusters which shared functional annotations. Finally, we added the genes *HO* and *ADE2* as negative and positive controls, respectively, for functionally dependent adaptation.

We also attempted to sort genes hit by newly acquired mutations into functionally related groups. To do this we developed an automated clustering criterion based only on correlations in genetic interaction profiles. Specifically, we used a genetic interaction profile correlation threshold of 0.2 to connect functionally similar genes into groups we call “interaction clusters”.

### Constructing gene deletion founders and fitness assay reference

The strains used in this study are derived from yAN184, a haploid MAT**a** strain of the BY background with the genotype: *his3*Δ*1*, *leu2*Δ*0*, *met17*Δ*0*, *ura3*Δ*0*, *trp1*Δ*0*. We replaced the *HML* locus in yAN184 with *MET17* and inserted a fluorescently labeled mating type selection “Magic marker” *RPL39pr-ymCherry-tADH1-Ste2pr-SpHIS5-tSkHIS3-Ste3pr-LEU2* at the *CAN1* locus [38] to create yJIR4.

We next constructed our set of Founder gene deletion mutants from yJIR4 by replacing the gene to be deleted with a doubly counter-selectable cassette, *UWMX* (*pTEF-CaURA3-tADH1-pCgTRP1-CgTRP1-tTEF*). This cassette contains *URA3* from *Candida albicans* and *TRP1* from *Candida glabrata* flanked by the *TEF1* promoter and terminator sequences that are homologous to the *KanMX* cassette. To create the mutants, we amplified a *KanMX* cassette (along with 400 bp of both upstream and downstream DNA for homology) from the appropriate yeast deletion collection haploid strain [39] and co-transformed it with a NotI digest of pFA6a-UWMX (S3 Fig). The resulting transformants were selected on uracil and tryptophan dropout media, and replica plated to YPD G418 200 mg L^−1^(GoldBio #G-418-25) to ensure the desired product of recombination (between the *KanMX* amplicon and the genome and between the *UWMX* and *KanMX*, S4 Fig). For each gene of interest, we screened three transformants for correct cassette integration by PCR following the yeast deletion collection protocol [39]. We were able to construct and verify 37 of the 45 deletion mutants we attempted.

To create a reference strain for competitive fitness assays, we replaced *ymCherry* with *ymEGFP* and inserted *UWMX* at the inactive *HO* locus in yJIR4 to create yJIR9. Except for the fluorescent marker, this strain has the same genotype as the *ho*Δ founder; it was used as a reference in all fitness assays.

### Experimental evolution

From each of the 37 gene deletion strains, we picked 20 independent colonies to found replicate populations. We propagated the resulting 740 populations in batch culture for 500 generations, using the experimental evolution protocol previously described by Lang et al. [40]. Briefly, we randomly arrayed the 740 populations across eight flat-bottom polypropylene 96-well microplates (Greiner, VWR catalog #29445-154). We randomly interspersed 28 blank wells to allow us to monitor potential cross-contamination events (no such events were observed). Each population was maintained in one well in 128 μL of rich laboratory media YPD (1% yeast extract (BD, VWR catalog #90000-722), 2% peptone (BD, VWR catalog #90000-368), and 2% dextrose (BD, VWR catalog #90000-904)), at 30°C. Each day, we resuspended populations by shaking at 1000 rpm for 2 min on a Titramax 100 plate shaker, and diluted them 1: 2^5^ twice using a BiomekFX liquid handling robot (Beckman Coulter). Every 100 generations, we added glycerol to each plate to a final concentration of 10% w/v, sealed the plates with an aluminum seal, and stored them at -80°C. This protocol results in approximately 10 generations per day at an effective population size of approximately 10^5^. Over the course of the experiment, 4 populations were lost due to pipetting error.

### Fitness assays

We measured the fitness of strains and evolved populations by direct competition to the fluorescently labeled reference strain yJIR9, using the protocol described previously by Jerison et al. [12]. Briefly, we revived frozen strains, populations, and references from frozen stocks by diluting them 1: 2^5^ into fresh YPD media. After 24 hours, the test strain or population was mixed 1:1 by volume with the reference and thereafter maintained using the same protocol used for evolution. 10 and 30 generations after mixing, the mixed populations were analyzed on the Fortessa or LSRII flow cytometers (BD Biosciences) to measure the ratio of reference to non-reference cells *r*. From these ratios, we calculated the fitness difference between the test and reference strains, *s*, given by $s=\frac{1}{t}\log(\frac{r_{f}}{r_{i}})$, where *r*_*f*_ and *r*_*i*_ are the final and initial measurements of the ratio of reference to non-reference, respectively, and *t* is the number of generations between those measurements.

### Reverting founding deletions

To revert the founding gene deletion mutations, we transformed an intact copy of the deleted gene (PCR amplified from BY4741 genomic DNA) and counter selected both the *URA3* and *TRP1* genes that were used to delete the original gene using standard protocols. The double counter selection dramatically increases the probability that the resulting transformants have the intended reversion, but these transformants also differ from their parents in being uracil and tryptophan auxotrophs. To correct for this discrepancy, we isolated three clones from each successful reversion and inserted our doubly counter selectable cassette *UWMX* at the neutral *HO* locus using a PmeI digest of the plasmid pHO-UWMX (S5 Fig), which has the *UWMX* cassette flanked by homology to *HO*. S6 Fig shows that the *UWMX* cassette had consistently positive effect across clones, though not always of the same magnitude.

Some clones proved impossible or nearly impossible to revert. In these clones, our double counter selection yielded no viable transformants. It is possible that some of these clones acquired mutations that affected the ability of clones to be transformed. Alternatively, it is also possible that a mutation changing the copy number of the counter selectable cassette has made reversion effectively impossible, as a successful reversion would require multiple integrations of the wild-type gene. Thus, tandem duplications, aneuploidy, or auto-diploidization (all common types of mutations observed in yeast laboratory evolution experiments [20, 21, 41–43]) would make reversions using our method impossible. We note that auto-diploidization is a particularly likely possibility since it has a beneficial fitness effect and can arise at high rates during laboratory evolution [20].

Since transformation may induce new mutations, we analyzed the fitnesses of replicate transformants for evidence of new mutations. All but 7 of the 100 clones produced 3 independent transformants with less than 0.5% standard deviation in fitness. The 7 clones which produced transformants of significantly different fitnesses all showed a clear pattern where only one of the three was significantly different from the median fitness (S1 Fig), as we would expect if transformation had induced a new mutation in that clone. Thus by using the median fitness of the three transformants in our analysis, we largely correct for these instances when transformation induced new mutations.

### Genome sequencing and analysis

We sequenced a clonal isolate from each of 100 populations whose founding deletion was successfully reverted, as well as the 37 gene deletion founders and yJIR4, the ancestor of the gene deletion founders. Indexed genomic DNA libraries were prepared as previously described [44] and sequenced on an Illumina NextSeq 500.

We first trimmed reads using trimmomatic v0.35 function ‘Illuminaclip’, with options: LEADING:3 TRAILING:3 SLIDINGWINDOW:4:15 MINLEN:36 [45]. We then used breseq v0.27.1 [23] to align the trimmed reads to the reference genome of the strain BY4741 from the University of Toronto [46]. All further analyses were conducted using the conservative list of mutation calls output by breseq. We note that while this approach does detect many small indels (on the order of 30 bps or less), it tends to miss larger indels and rearrangements. In principle, we could attempt to detect these events using a junction-calling approach, but given our limited sequencing depth in this study, we cannot accurately confirm calls based on coverage variation, so the potential for false positives is high. Similarly, due to limited coverage we cannot confidently call mitochondrial mutations. Thus to avoid excessive false positives, we have not attempted to call these types of events.

### Hierarchical models of fitness evolution and epistasis

To understand the different factors that contribute to the rate of adaptation, we inferred the parameters to a model analogous to model 3B in Kryazhimskiy et al. [10]. Briefly, each fitness measurement is one of 3 replicate measurements, *l*, of one of 20 replicate evolved populations, *k*, descended from one of 37 gene deletion founders, *j*, which belongs to one of 16 modules, *i*. In our model, we assume that each measurement of the change in fitness of each population after 500 generations, *y*_*ijkl*_, is the sum of an average fitness across all populations, *α*, a linear effect of initial fitness, *βx*_*ij*_, a Founder module specific random effect, *m*_*i*_, a Founder gene specific random effect, *g*_*ij*_, a population specific random effect (which accounts for evolutionary stochasticty), *p*_*ijk*_, and a term to account for measurement error, *τ*_*ijkl*_:

$$\begin{array}{c} y_{ijkl}=\alpha +\beta x_{ij}+m_{i}+g_{ij}+p_{ijk}+\tau_{ijkl}\\ m_{i}∼N(0,\sigma_{m}^{2})\\ g_{ij}∼N(0,\sigma_{g}^{2})\\ p_{ijk}∼N(0,\sigma_{p}^{2})\\ \tau_{ijkl}∼N(0,\sigma_{\tau }^{2}). \end{array}$$

The maximum likelihood parameter values for this model are summarized in the last of row Table 3 and the corresponding variance components are summarized in Fig 2C. Finally, to investigate the significance of the different factors, we compared the model above to the nested set of simpler models using the likelihood ratio test. The maximum likelihood fits for all models are shown in Table 3. In all comparisons, the likelihood ratio test rejects the less complex model.

**Table 3:** Models of fitness evolution.

	*α*	*β*	*σ*_*m*_	*σ*_*g*_	*σ*_*p*_	*σ*_*τ*_	*ℓ*
*α* + *g*_*ij*_ + *p*_*ijk*_	4.96	—	—	4.29	1.45	0.81	-2833
*α* + *m*_*i*_ + *g*_*ij*_ + *p*_*ijk*_	5.40	—	3.83	2.47	1.45	0.81	-2828
*α* + *βx*_*ij*_ + *g*_*ij*_ + *p*_*ijk*_	1.35	-0.42	—	3.04	1.45	0.81	-2823
*α* + *βx*_*ij*_ + *m*_*i*_ + *g*_*ij*_ + *p*_*ijk*_	2.01	-0.39	2.47	1.78	1.45	0.81	-2817

Maximum likelihood parameter values for the hierarchical models of the fitness increment. Each model is denoted by its expected fitness increase after 500 generations. In all comparisons of nested models, the likelihood ratio test rejects the less complex model.

To understand the different factors that contribute to epistasis between founder gene deletion and evolved mutations, we inferred the parameters of an analogous hierarchical model. In this model, epistasis between evolved mutations and the Founder deletion mutations is a function of the fitness effects of the founder gene deletions on the wild-type background along with the random effects described above. The maximum likelihood fits and analysis of nested models is summarized in Table 4.

**Table 4:** Models of epistasis between founder gene deletions and evolved mutations.

	*α*	*β*	*σ*_*m*_	*σ*_*g*_	*σ*_*p*_	*σ*_*τ*_	*ℓ*
*α* + *g*_*ij*_ + *p*_*ijk*_	3.85	—	—	7.23	2.66	0.49	-479
*α* + *m*_*i*_ + *g*_*ij*_ + *p*_*ijk*_	4.89	—	7.56	0.00	2.71	0.49	-463
*α* + *βx*_*ij*_ + *g*_*ij*_ + *p*_*ijk*_	-1.88	-0.73	—	4.42	2.65	0.49	-465
*α* + *βx*_*ij*_ + *m*_*i*_ + *g*_*ij*_ + *p*_*ijk*_	0.44	-0.58	4.39	0.00	2.56	0.49	-450

Maximum likelihood parameter values for the hierarchical models of epistasis. Each model is denoted by its expected epistasis. In all comparisons of nested models, the likelihood ratio test rejects the less complex model.

### Mutual information analysis between founder genotypes and evolved mutations

To measure the association between evolved mutations and founder genotypes, we used a test statistic based on mutual information similar to the one described in Jerison et al. [12]. Briefly, we define the mutual information between the possible modules or genotypes of a founder, *W* ∈ {*W*_1_, …, *W*_*m*_}, and the possible multi-hit genes or multi-hit modules of evolved mutations *g* ∈ {*g*_1_, …, *g*_*n*_}, as:

$$\begin{array}{c} M\left(W,g\right)=\sum_{g=(g_{1},\ldots ,g_{n})}\sum_{W=(W_{1},\ldots ,W_{n})}p\left(W\right)\sum_{m_{g}=\left(0,1\right)}p\left(m_{g}|W\right)\log_{2}\frac{p(m_{g}|W)}{p(m_{g})}, \end{array}$$

where *m*_*g*_ is an indicator variable with value 1 when an evolved mutation belongs to *g* and 0 otherwise. We estimate probabilities from observed counts: *p*(*W* = *W*_*i*_) is the frequency of populations with property *W*_*i*_, *p*(*m*_*g*_ = 1) is the frequency of populations with a mutation in *g* across all populations, and *p*(*m* = 0|*W* = *W*_*i*_) is the frequency of populations without a mutation in *g* among populations with property *W*_*i*_.

To measure the additional mutual information provided by a property of the founders after accounting for a second property, *Z*, we use conditional mutual information defined as:

$$\begin{array}{c} M\left(W,g|Z\right)=\sum_{g}\sum_{Z}p\left(Z\right)\sum_{W}p\left(W|Z\right)\sum_{m_{g}}p\left(m_{g}|W,Z\right)\log_{2}\frac{p(m_{g}|W,Z)}{p(m_{g}|Z)}. \end{array}$$

We calculate this statistic for founder modules with evolved genes and founder modules with evolved modules, as well as for founder genes with evolved genes and evolved modules, conditioning on founder module. Then, we compare these statistics to null distributions generated by permuting mutations across populations, keeping the number of mutations per population fixed. We report the mutual information in excess of null, $M\left(\cdot \right)-{\bar{M}}_{p}\left(\cdot \right)$, with 95% confidence intervals calculated from the null distribution (Table 2).

## Supporting information

S1 Fig Mutations acquired during transformation. Difference from the median fitness of 3 independent transformants of 100 reverted clones arranged arbitrarily on the x-axis. Transformants descended from the same clone are connected by a gray line. The outliers always show the same pattern of two transformants with nearly equal fitness and one mutant.
(PDF)

S2 Fig Compensatory of evolution of *ade*Δ. Schematic of a subset of the adenine biosynthesis patway showing the causal order of relevant genes. For simplicity, only relevant metabolites are labeled. Abbreviations: AIR, 5′-phosphoribosylaminoimidazole; CAIR, 5′-phosphoribosylaminoimidazole carboxylate. The red cross indicates the founding gene deletion and the blue hashes indicate independently acquired mutations in populations descended from the *ade2*Δ Founder.
(PDF)

S3 Fig Map of pFA6a-UWMX. See S3 Data.
(PDF)

S4 Fig Diagram showing double recombination construction of founders. To delete the gene *YFG* we co-transform the *KanMX* gene amplified from the appropriate deletion collection strain so as to contain homology upstream and downstream of *YFG* and our *UWMX* gene purified from pFA6a-UWMX.
(PDF)

S5 Fig Map of pHO-UWMX. See S4 Data.
(PDF)

S6 Fig Fitness effect of counter selectable cassette. Fitness of 100 reverted clones plotted against their fitness after adding the counter selectable cassette *UWMX*.
(PDF)

S7 Fig Number of fixed nonsynonymous mutations. Each point represents the number of fixed nonsynonymous mutations in an evolved population. Populations are ordered according to (A) the initial fitness effect of their founding gene deletion or (B) the fitness gain acheived by the population. Refer to Fig 2 for the symbol legend.
(PDF)

S8 Fig Modular epistasis in Szamecz et al. [11]. (A) Relationship between initial fitness of the 187 Founder gene deletion mutants and the mean fitness gain of the 4 replicate populations descended from that Founder after approximately 400 generations of evolution. Founders colored according to interaction cluster with unclustered Founders in black (see S5 Data). (B) Fraction of the variance between populations in fitness gain after 400 generations of evolution that is attributable to each indicated component. (Note that we were not able to estimate the contribution of measurement error since only one measurement was available for each population.)
(PDF)

S1 Table Within module correlations. Mean correlation in genetic interaction profiles between genes in each module. Correlations from Data file S3 in Costanzo et al. [3].
(TSV)

S2 Table Reversibility of gene deletion mutations in evolved populations. Successful reversions/attempts for evolved populations descended from different gene deletion mutants.
(TSV)

S3 Table List of multi-hit interaction clusters. Interaction clusters that were independently mutated in at least two different populations. The Ras/cAMP and mating pathways (interactions clusters 1 and 2, respectively) were assigned by hand since neither pathway is well represented in the genetic interaction map.
(TSV)

S1 Data Fitness measurements for all populations after 500 generations of evolution. (TSV)

S2 Data Coding mutations acquired by clones in a subset of 100 populations. (TSV)

S3 Data Annotated sequence of pFA6a-UWMX. (GB)

S4 Data Annotated sequence of pHO-UWMX. (GB)

S5 Data Interaction clusters for Szamecz et al. [11]. (TSV)
