# Supplementary figures and images for: Modular epistasis and the compensatory evolution of gene deletion mutants
Source: PLoS Genet. 2019 Feb 15;15(2):e1007958. doi: 10.1371/journal.pgen.1007958 (PMC6395002; doi:10.1371/journal.pgen.1007958)

Difference from median fitness (%)

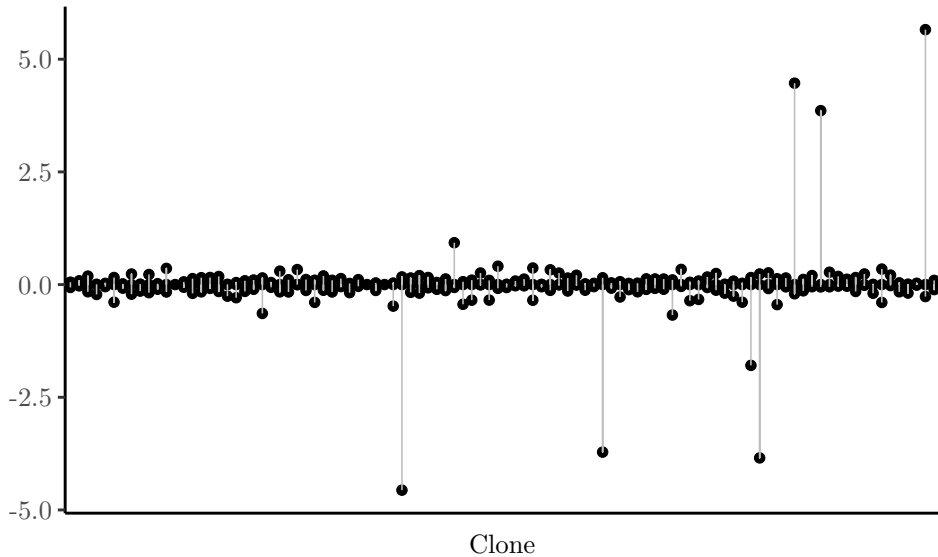

Supplement: S1 Fig — Difference from the median fitness of 3 independent transformants of 100 reverted clones arranged arbitrarily on the x-axis. Transformants descended from the same clone are connected by a gray line. The outliers always show the same pattern of two transformants with nearly equal fitness and one mutant. (PDF) [file pgen.1007958.s001.pdf]

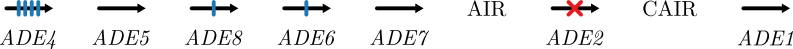

Supplement: S2 Fig — Schematic of a subset of the adenine biosynthesis patway showing the causal order of relevant genes. For simplicity, only relevant metabolites are labeled. Abbreviations: AIR, 5′-phosphoribosylaminoimidazole; CAIR, 5′-phosphoribosylaminoimidazole carboxylate. The red cross indicates the founding gene deletion and the blue hashes indicate independently acquired mutations in populations descended from the ade2Δ Founder. (PDF) [file pgen.1007958.s002.pdf]

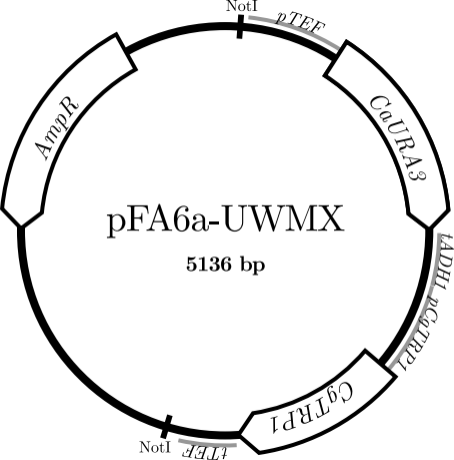

Supplement: S3 Fig — See S3 Data. (PDF) [file pgen.1007958.s003.pdf]

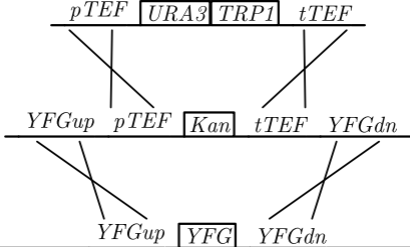

Supplement: S4 Fig — To delete the gene YFG we co-transform the KanMX gene amplified from the appropriate deletion collection strain so as to contain homology upstream and downstream of YFG and our UWMX gene purified from pFA6a-UWMX. (PDF) [file pgen.1007958.s004.pdf]

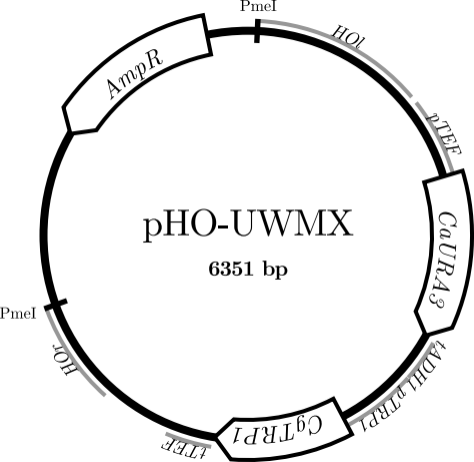

Supplement: S5 Fig — See S4 Data. (PDF) [file pgen.1007958.s005.pdf]

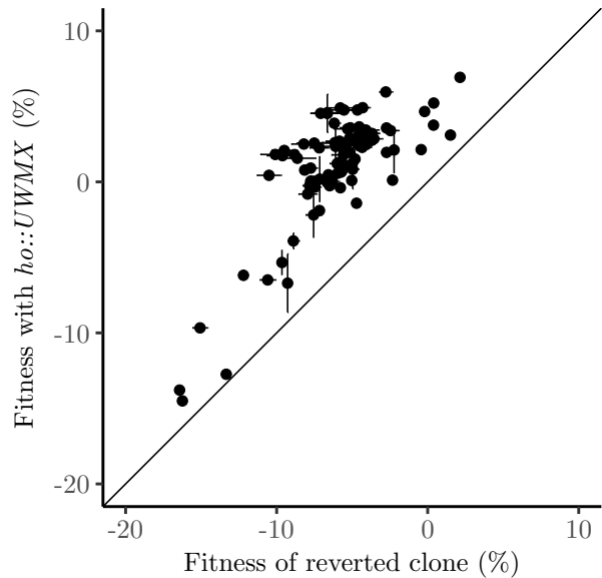

Supplement: S6 Fig — Fitness of 100 reverted clones plotted against their fitness after adding the counter selectable cassette UWMX. (PDF) [file pgen.1007958.s006.pdf]

A

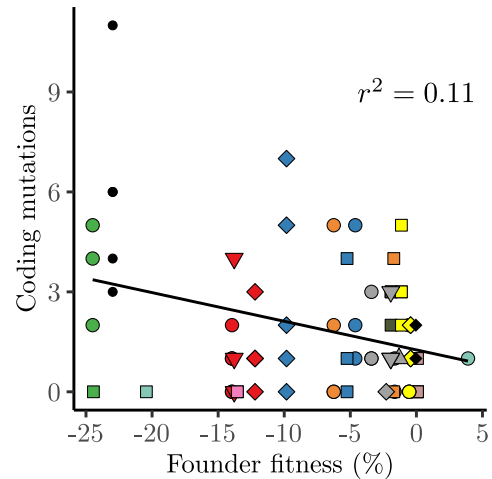

B

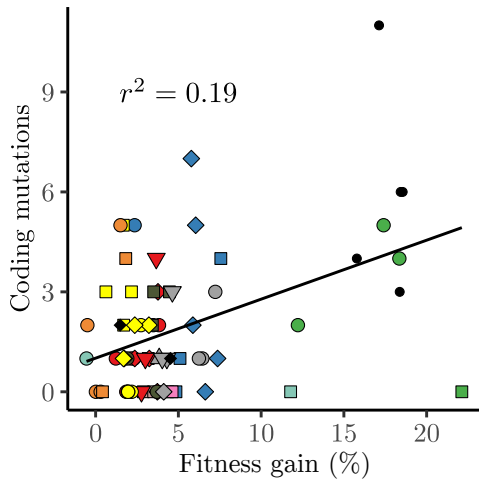

Supplement: S7 Fig — Each point represents the number of fixed nonsynonymous mutations in an evolved population. Populations are ordered according to (A) the initial fitness effect of their founding gene deletion or (B) the fitness gain acheived by the population. Refer to Fig 2 for the symbol legend. (PDF) [file pgen.1007958.s007.pdf]

A

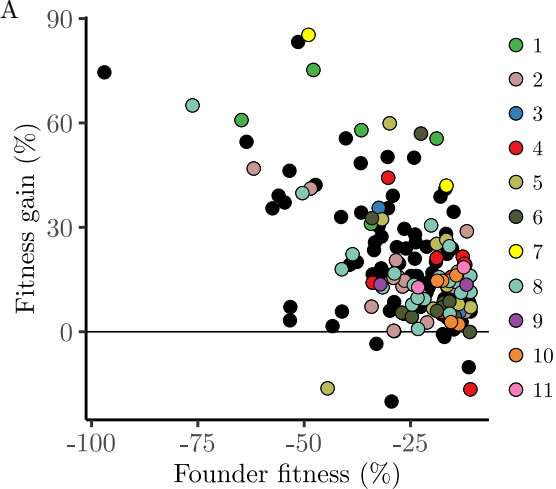

B

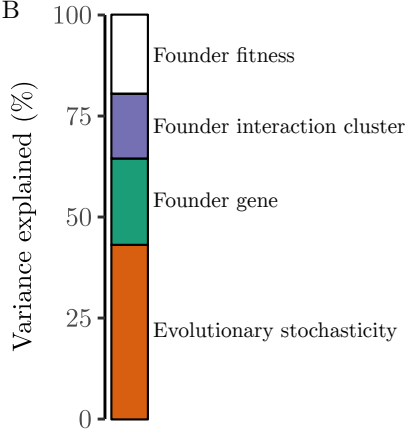

Supplement: S8 Fig — (A) Relationship between initial fitness of the 187 Founder gene deletion mutants and the mean fitness gain of the 4 replicate populations descended from that Founder after approximately 400 generations of evolution. Founders colored according to interaction cluster with unclustered Founders in black (see S5 Data). (B) Fraction of the variance between populations in fitness gain after 400 generations of evolution that is attributable to each indicated component. (Note that we were not able to estimate the contribution of measurement error since only one measurement was available for each population.) (PDF) [file pgen.1007958.s008.pdf]
